# SAMstrt: statistical test for differential expression in single-cell transcriptome with spike-in normalization

**DOI:** 10.1093/bioinformatics/btt511

**Published:** 2013-08-31

**Authors:** Shintaro Katayama, Virpi Töhönen, Sten Linnarsson, Juha Kere

**Affiliations:** ^1^Department of Biosciences and Nutrition, Karolinska Institutet, 141 83 Huddinge, Sweden, ^2^Science for Life Laboratory, Karolinska Institutet Science Park, 171 21 Solna, Sweden and ^3^Department of Medical Biochemistry and Biophysics, Karolinska Institutet, 171 77 Stockholm, Sweden

## Abstract

**Motivation:** Recent transcriptome studies have revealed that total transcript numbers vary by cell type and condition; therefore, the statistical assumptions for single-cell transcriptome studies must be revisited. SAMstrt is an extension code for SAMseq, which is a statistical method for differential expression, to enable spike-in normalization and statistical testing based on the estimated absolute number of transcripts per cell for single-cell RNA-seq methods.

**Availability and Implementation:** SAMstrt is implemented on R and available in github (https://github.com/shka/R-SAMstrt).

**Contact:** shintaro.katayama@ki.se

**Supplementary Information:** Supplementary data are available at *Bioinformatics* online.

## 1 INTRODUCTION

Unbiased molecular measurements are required to form precise hypotheses on molecular mechanisms, and measurements at single-cell resolution can give detailed snapshots of heterogeneous cell cultures or tissues. Recently described single-cell mRNA sequencing technology is one promising method for such purposes (Hashimshony *et al.*, 2012; Islam *et al.*, 2012; Ramsköld *et al.*, 2012; Tang *et al.*, 2011). However, the statistical assumptions in the traditional tests for differential expression are not necessarily applicable, as the total mRNA molecules per cell could be different by cell types (Dobson *et al.*, 2004; Islam *et al.*, 2011; Lovén *et al.*, 2012), and sensitivity over the experiments is less consistent than in the other methods that use large amounts of input mRNA. One possible solution is the addition of a known number of spike-in control RNA molecules to each cell lysate, which can be used to normalize and convert read counts to (estimated) molecule numbers. In the present study, we used the single-cell tagged reverse transcription (STRT) method (Islam *et al.*, 2012) to investigate key issues for statistical tests, in several control experiments, and then implement and demonstrate a new concept for differential expression test on single-cell transcriptome profiles.

## 2 ISSUES FOR STATISTICAL TESTING IN SINGLE CELLS

**Fig. 1.**
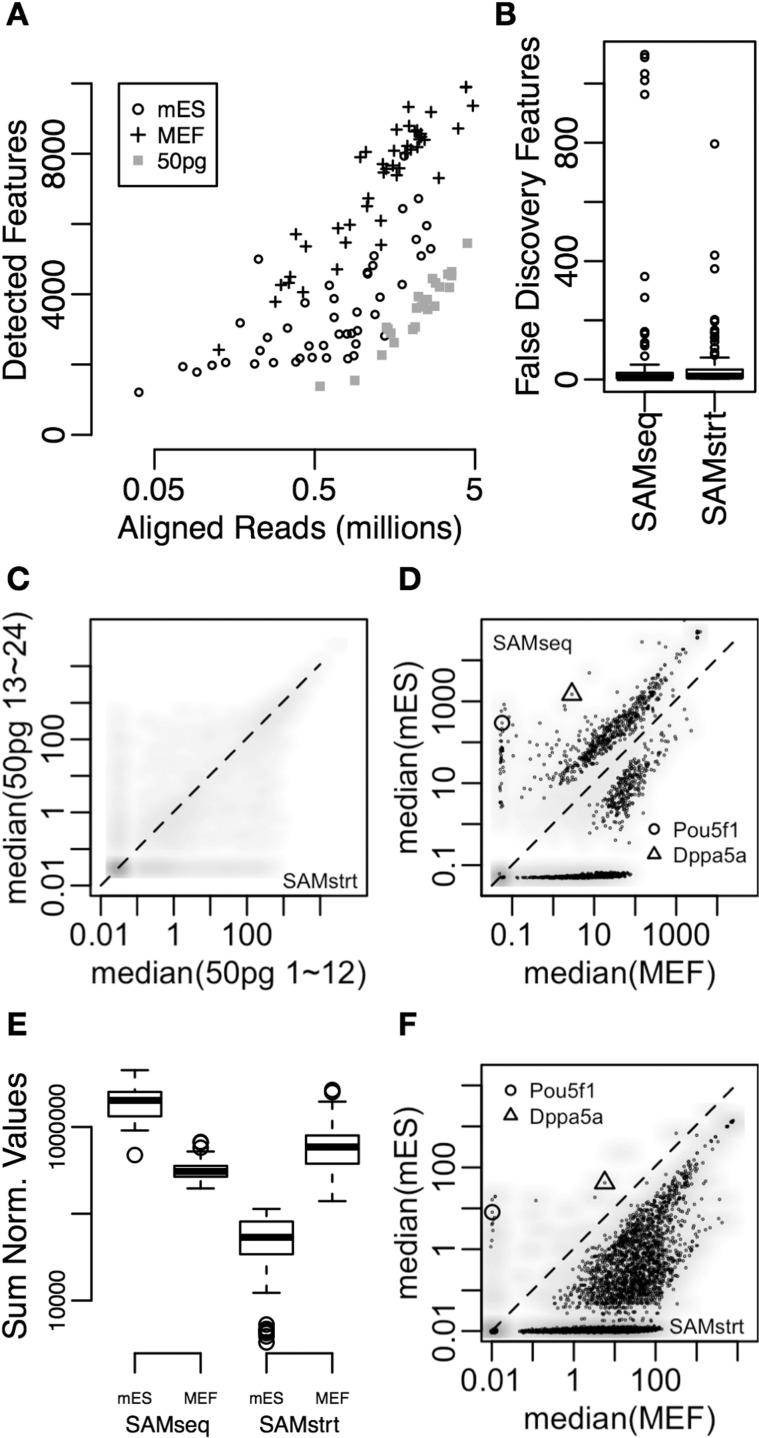
(**A**) Aligned reads and the detected features in 45 mES, 44 MEF and 24 of 50 pg human brain total RNAs (50 pg) by STRT. The features are known genes, repeat elements and spike-in molecules; there were 25 286 features for mouse and 25 665 features for human. (**B**) False discovery features in 100 trials between SAMseq and SAMstrt. In each trial, 24 of 50 pg human brain total RNAs were separated randomly into two groups, and then each method compared the two groups. Although number of differentially expressed features should be zero in all trials if no technical variations, the false discovery features showed statistical difference (FDR < 1%). (**C**) Comparison of transcripts per 50 pg human brain total RNAs per feature, between the former 12 samples and the latter 12 samples. This is one representative comparison in the trials for panel B, and there were no significantly different features by the proposed SAMstrt (FDR < 0.01%). Gray color gradation denotes density of features in the scatter plot, and expression level of each feature is represented by median. Dashed diagonal line denotes equivalent expression between two samples. (**D**) Comparisons of normalized expression levels per cell per feature, between 45 mES and 44 MEF cells, by SAMseq. Usage of the gray color gradation and the dashed diagonal line are same with the panel C. Points are differentially expressed features (FDR < 0.01%). (**E**) Sum of the normalized expression values of all features by samples and the comparison by the methods. (**F**) Comparison of transcripts per cell per feature, between 45 mES and 44 MEF cells, by SAMstrt. Usage of the gray color gradation, the dashed diagonal line, and the points are same with the panel D. SAMseq added uniform random numbers between 0 and 0.1 to all values to avoid ties (Li and Tibshirani, 2011), therefore, features which are the most bottom expression level in the panel C, D and F are no expression, or less than detection limit. The normalized expression values by SAMstrt at the panel C and F are moreover estimated transcripts per cell based on the initial concentration of the spike-in molecules

There are two challenges to be aware of for the statistics of STRT single-cell transcriptome profiling. The first issue is the variation of sequencing depth and aligned read counts. STRT sequencing of mouse embryonic stem cells (mES) and fibroblasts (MEF) in the earlier study (Islam *et al.*, 2011) had about 100-fold differences in the sequencing depths (Fig. 1A). In our 24-plexed sequencing of 50 pg pooled human brain total RNA, there was only 10-fold difference in the depths (Fig. 1A). Also mES cells tended to have less aligned read numbers in comparison with MEFs (Fig. 1A), as the mES cells express less mRNA (Islam *et al.*, 2011). Even when we consider the existence of true biological variation in the cells by transcriptional bursting (Chubb *et al.*, 2006; Raj *et al.*, 2006) or heterogeneity of the cultured cells, it is obvious that variation of the sequencing depth is a contributing cause for the variation of sensitivity; e.g. experiments at shallow sequencing depth could detect a smaller number of features (Fig. 1A). Equalization of RNA or non-amplified cDNA amounts before sequencing was not feasible, as barcoded cDNAs synthesized from each cell were pooled before amplification without any control of molecular concentration (Islam *et al.*, 2012).

The second issue to consider is the process and underlying hypothesis for normalization. Normalization is required to correct bias coming from technical sources. For statistical tests of differential expression in sequencing-based profiles, the most recent proposed normalization procedures include rescaling by estimated sequencing depth (Anders and Huber, 2010; Bullard *et al.*, 2010; Li and Tibshirani, 2011; Robinson and Oshlack, 2010), and those are performed under the assumption that the majority of co-expressing genes are not differentially expressed between two samples. This hypothesis is relevant in cases where we use equivalent amount of RNAs, cDNAs or sequence templates as for usual RNA-seq, but it would be inappropriate for single-cell transcriptome sequencing where the underlying biological heterogeneity may include heterogeneity of the total transcript count between cells.

To handle these two issues, we have developed a method for robust statistics that has tolerance for large sequencing depth variations and that can also perform normalization that considers differences of total transcript counts per cell.

## 3 CONCEPT AND VALIDATION

We adapted SAMseq (Li and Tibshirani, 2011) to test for differential expression in STRT single-cell transcriptome profiles. The framework of this method is Poisson resampling and non-parametric statistics, which yield robust statistics even in huge variation of sequencing depths, as the authors demonstrated for SAMseq. We developed this further and modified the sequencing depth estimation by assuming equivalent spike-in-molecules/cell in each experimental set. One concern is the abundance of the spike-in molecules among the sequenced reads. Although it would vary by target cell types (e.g. large cells may have more mRNA molecules), the amount of the spike-in molecules is usually at most 1% of total poly-A+ molecules in each sequencing reaction, and also counts of the control molecules fluctuate because of technical variation. To estimate the feasibility of this approach, we separated the 24-plex trial sequencing results into two groups randomly and then tested the differential expression by both sequencing depth estimations. As this is repetitive sequencing of the pooled RNA, ‘no difference’ is the ideal result; however, a few features would be recognized as differentially expressed owing to the technical variation. Actually, 11 features were unexpectedly recognized as differentially expressed by the spike-in-based normalization, whereas six by the original (Fig. 1B; the values are median in the repetitive trials; 46.44 and 76.01 features in average, respectively; there were 25 665 defined and 15 192 detected features in total of the trial results). As there were no significant differences between two methods (*P* = 0.2394 by Welch’s two sample *t*-test for the distributions) and both methods kept a few false discovery in the trials (e.g. Fig. 1B and C), median representation of the technical replicas gives an impression of differential expression between the two groups, when there were no significant differences because of broad and random variations over the replicas. Thus, we conclude that the spike-in-based normalization works when given enough sequence spike-in reads; there were 5000 spike-in reads on average in the trial results.

We then confirmed our approach by comparison of actual cells as well, between the mES and the MEF. As mES cells express less mRNA than MEF (Islam *et al.*, 2011), many genes in mES cells should yield also less transcripts/cell in comparison with MEF cells (right lower diagonals of Fig. 1D and F) However, the original SAMseq judged that there were 598 highly expressed features in mES cells significantly (FDR < 0.01%; Fig. 1D). One possible reason is overestimation of mES expression levels by the original method (Li *et al.*, 2011), which is normalization by putative stably expressed endogenous features. Although the mES cells with less mRNA had fewer aligned reads and fewer detected features (Fig. 1A), actually the original method increased the estimated mRNA content of mES cells (Fig. 1E) because the original normalization assumes that the majority of co-expressing genes are not differentially expressed. However, it contradicts our validated observations (Islam *et al.*, 2011). In contrast, the proposed SAMstrt judged that there were only 15 highly expressed features in mES cells, whereas there were 8441 less expressed features (Fig. 1F). However, the sum of normalized reads was less in mES cells than MEF (Fig. 1E), as we had observed (Islam *et al.*, 2011). Moreover, well-known ES cell markers, e.g. *Pou5f1* and *Dppa5a*, were still among the significantly accumulated genes in mES.

These results lead to the conclusion that the spike-in normalization yields accurate quantitation and sensible statistical tests. For example, if a mononuclear-cell A contained 100 000 transcripts, a mononuclear-cell B contained 400 000 transcripts and a gene expressed 100 transcripts in both cells, the proposed method would avoid to detect it as differentially expressed. We consider that as a valid interpretation, as there was no change of the transcript count per nucleus between those two cells. Instead, there must have been (many) other genes that were overexpressed in cell B.

## 4 IMPLEMENTATION

The proof-of-concept was implemented in R (version 3.0) with the samr package (version 2.0). Presently, users need to install this package by R CMD install with a downloaded file from https://github.com/shka/R-SAMstrt/archive/0.99.0.tar.gz followed by installation of samr. However, this package will be in the future bioconductor releases (submitted), and the bug-fix and new features will be released at github and bioconductor. This package is released under the GNU Lesser General Public License (LGPL) version 3. Although the help documents of the package contain basic usages, Supplementary Data file of this article includes more concrete sample codes and the input data for Figure 1D and F.

It is necessary to prepare a SAMseq-compatible feature matrix as input, and the matrix must contain several rows beginning with ‘RNA_SPIKE_’ (e.g. RNA_SPIKE_1). Those are read counts, which aligned to the spike-in sequences. Therefore, users can apply this package to any RNA-seq libraries using not only 8 ArrayControl spike-in molecules but also, for example, 92 ERCC spike-ins.

This implementation contains two functions; one is a statistical test for differential expression, and the other one is calculation of the normalized values. Loading of the SAMstrt package [library(SAMstrt)] overwrites the sequencing-depth estimation of SAMseq for the spike-in normalization. Therefore, users can use all problem types (e.g. comparisons of more than two groups, or paired comparison of two groups) and the other optimization parameters also for the spike-in-based statistical tests via function SAMseq after loading of the SAMstrt package.

SAMstrt.normalization calculates the spike-in-based normalized values with conversion to estimated transcripts/sample values based on the initial concentration of spike-in RNAs. Those estimations would be transcripts/cell in single-cell experiments and would be transcripts/1 ng-totalRNA in case of 1-ng experiment; however, users should count number of cells before RNA extraction for the latter case. This function would be useful for visualization of the expression changes, or the other type of statistics, e.g. principal component analysis. However, this package does not normalize mRNA length bias (neither does SAMseq), as theoretically STRT synthesizes only one first-strand cDNA from one poly(A)+ RNA (Islam *et al.*, 2012), whereas full-length RNA-seq expects several fragmented cDNAs from one RNA molecule with the number of fragment correlating to the length of the original RNA molecule. Moreover, neither this package nor the original SAMseq normalizes GC-content bias of the sequencing templates. Therefore, additional experimental (e.g. incorporation of unique molecular identifiers into the first-strand cDNAs before amplification, Kivioja *et al.*, 2012) or mathematical normalization before the spike-in-based normalization would improve accuracy of the estimation and the statistical tests.

In conclusion, this proof-of-concept package SAMstrt would be applicable to user’s own results by STRT and other sequencing-based transcriptome profiling methods, including usual RNA-seq, with spike-in control molecules. This package with highly multiplexed library preparation enables flexible experimental designs and more accurate interpretations of differential expression, especially to reveal complex behaviors between many cells at single-cell resolution.

*Funding*: Karolinska Institutet Distinguished Professor Award (to J.K.); and the Strategic Research Programme in Diabetes at Karolinska Institutet. The computations were performed on resources provided by SNIC through Uppsala Multidisciplinary Center for Advanced Computational Science (UPPMAX) under Project b2010037.

*Conflict of Interest*: none declared.

## Supplementary Material

Supplementary Data
