# Supplementary material for: SAMstrt: statistical test for differential expression in single-cell transcriptome with spike-in normalization
Source: Bioinformatics. 2013 Aug 31;29(22):2943–5. doi: 10.1093/bioinformatics/btt511 (PMC3810855; doi:10.1093/bioinformatics/btt511)
Supplement: Supplementary Data [file supp_29_22_2943__index.html]

SAMstrt: Statistical test for differential expression in single-cell transcriptome with spike-in normalization — SAMstrt: statistical test for differential expression in single-cell transcriptome with spike-in normalization — SAMstrt: statistical test for differential expression in single-cell transcriptome with spike-in normalization — Supplementary Data 

# SAMstrt: statistical test for differential expression in single-cell transcriptome with spike-in normalization

## Supplementary Data

files

**Files in this Data Supplement:**

- Supplementary Data - zip file
